# Effect of pinch types on pinch force sense in healthy adults

**DOI:** 10.3389/fnhum.2022.990431

**Published:** 2022-10-26

**Authors:** Lin Li, YanXia Li, Peng Jia, Shuyan Wang, Wanpeng Wang, Yuxiang Liu

**Affiliations:** ^1^Department of Physical Education, Renmin University of China, Beijing, China; ^2^College of Physical Education, Langfang Normal University, Langfang, Hebei, China

**Keywords:** pinch, force sense, sex, proprioception, force reproduction task

## Abstract

Pinch force sense plays an important role in the performance of daily finger movements, including tip, key, palmar pinch. The present study investigated the roles of pinch type in the sensation of pinch force among healthy participants in the ipsilateral force reproduction trial. This study instructed forty healthy adult subjects (20 women and 20 men) in producing reference forces at different levels [10, 30, 50% maximal voluntary isometric contraction (MVIC)] by adopting 3 pinch types (tip, key, and palmar pinches) and in reproducing the above force levels with the identical hand. Our study revealed that subjects are significantly more sensitive detecting alterations of pinching forces with tip pinch but not key or palmar pinch under high forces (30 and 50% MVIC) but not at lower force levels (10% MVIC).

## Introduction

Diverse pinch grip types (tip, key, or palmar pinch) or their different combinations at diverse force levels have usually been utilized at working sites (Mathiowetz et al., 1984, 1985; Puh, 2010; Tajika et al., 2015; Romero-Guzman et al., 2016; Maleki-Ghahfarokhi et al., 2019). Workers of diverse jobs, such as engineers, repairpersons or mechanics, should keep different pinch grips at steady, submaximal force levels by adopting different equipment or hand tools during various operations, ranging from small electronic part assembly to large aeroplane assembly. Spontaneous pinch on one object represents a complicated motor activity because a pinch force large enough should be used for preventing object from slipping (Blennerhassett et al., 2006; Iyengar et al., 2009; Ambike et al., 2014). However, excess force is unfavorable to avoid object crushing or unnecessary fatigue. In previous studies, repeat and excessively large pinch forces accounted for a risk factor for musculoskeletal disorders (MSDs) (Birkbeck and Beer, 1975; Feldman et al., 1983; Silverstein et al., 1986, 1987). To further understand the common factors leading to MSDs and establish preventive measures for decreasing injury incidence, it is necessary to understand different types of pinch grips. However, different pinch types may be used to operate the same device. The study of the force sense of different pinch types may be helpful for the design of tools that require precise force control. Previous studies have demonstrated that pinch types affect an individual’s maximal pinch strength (Imrhan and Loo, 1989; Shih et al., 2003; Jansen et al., 2008; Puh, 2010). Altered angular motion at the individual joints of the thumb and index finger are found in different pinch types (Li and Nimbarte, 2006). Therefore, there may be different force senses between different pinch types. To achieve a suitable pinch grip, it is necessary to precisely harmonize fingers by combining force control and sensation. With the growing number of fingers (key and palmar pinch), difficulties related to pinch grip harmonization increase (Kong et al., 2011; Kim et al., 2020). Additionally, the tip pinch has been reported to be the most precise grasp for small objects (Cutkosky, 1989; Dong et al., 2006). Hence, we hypothesize that the force sense of the tip pinch was more accurate than that of the key and palmar pinch.

It was necessary to investigate the pinch force perception in women, as increasingly more women have entered the workforce (Liao, 2014). Sex effects in controlling submaximal forces in force reproduction or a matching task are considered. Bao compared the predicted force with the known palmar pinch force and reported no significant effect of sex on reproducing force validity (Bao and Silverstein, 2005). Nonetheless, Herring-Marler studied the effect of adult sex on finger precision in matching the submaximal ramped force. The author discovered that the capacity of target force matching was significantly different, and males had increased errors compared with females (Herring-Marler et al., 2014). Similar to Herring-Marler, Shinohara et al. (2003), men are variable by four-finger (the index, middle, ring, and little fingers) ramp force production tasks. In addition, another study on handgrip force sense showed that women replicated force more accurately than men just at higher force levels (90–130 N) (Li et al., 2022). However, according to another report, females had markedly increased force-matching errors with 70° knee joint extension compared to males (Song et al., 2006). Apart from exhibiting muscle strength (Hallbeck et al., 1992; Bao and Silverstein, 2005; Herring-Marler et al., 2014), women have been reported to have an increased type-I fiber proportion (Simoneau and Bouchard, 1989), increased half-relaxation time (Hicks and McCartney, 1996), and decreased whole-muscle twitch force (Miller et al., 1993) compared with men. The difference in body size between male and female adults can be associated with the efficient gross motor performance in the former. However, fine motor activities demand distinct sensorimotor properties other than power and strength. Females have good performance in sensory discrimination-requiring activities (Herring-Marler et al., 2014). Females also have more type-I fibers; as a result, they can activate a greater amount of motor units with smaller forces than males do and perform well in modifying fine motor forces (Herring-Marler et al., 2014). Hence, we hypothesized that women reproduce pinch force more accurately and consistently than men.

To the best of our knowledge, there is no prior work assessing the relation of pinch type with pinch force sense. There are inconsistent reports regarding sex’s impact on errors in force reproduction. Consequently, the present work focused on investigating how sex and pinch types affected error in pinch force reproduction accuracy among healthy people.

## Materials and methods

### Subjects

This work tested forty healthy subjects (20 women and 20 men, aged 22.3 ± 6.5 years, body mass 64.0 ± 13.2 kg, height 169.2 ± 8.3 cm). The subjects utilized their right hand in writing, so they were deemed as right-handed. The adults presented no neuromuscular disorders and were naive to the task. All adults provided written informed consent. The current study was approved by Renmin University of China’s Ethics Review Board (reference number 2022012701). This work was completed in accordance with the Helsinki Declaration.

### Apparatus

The electronic digital force dynamometer (pinch analyzer; Kjyl Technologies, China, range of measurement: 0–150N, accuracy: 0.1%) was utilized to estimate force production and conduct strength tests. The manufacturer was responsible for instrument calibration. Error was maximally prevented by prior testing. In force reproduction estimation tests and strength tests, we set the dynamometer’s pinch span at 2 cm. The outputs from the electronic digital force dynamometer were converted and amplified by a A/D Converters (AD7190, ADInstruments, Bella Vista, NSW, Australia) and low-pass filtered at 10 Hz (Butterworth, fourth-order, zero-phase lag). This work set sampling frequency at 100 Hz. Based on the pinch analyzer, a protocol was developed that measures pinch force sense. Based on the high values of the intraclass correlation coefficient (ICC), the tip pinch (0.783–0.895) and palmar pinch (0.752–0.903) force sense tests demonstrated good reliability for all the variables. The ICCs for the key pinch (0.712–0.881) indicated fair to good relative test-retest reliability (Li et al., 2020).

### Protocol

**FIGURE 1:**
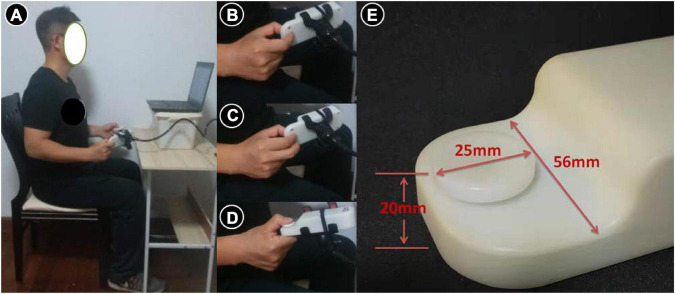
The standardized positioning **(A)** used for tip **(B)**, palmar **(C)** and key pinch **(D)** force sense measurement and the size **(E)** of the electronic digital force dynamometer.

The study was performed in a quiet room to ensure proper minimization of auditory distractions (Lubiatowski et al., 2013). All participants were instructed to sit on a chair approximately 60 cm ahead of the computer monitor. According to the guidelines from the American Society of Hand Therapists, the participants were supposed to have a systemic posture (Figure 1A), with vertical positioning of the upper arm, a 90°-flex of the elbow, and neutral positions of the wrist and forearm (van den Noort et al., 2014). Participants carried out the isometric pinching tasks using 3 pinch types: tip, palmar, and key pinches. Tip pinch (Figure 1B): thumb pad to index pad, and additional fingers were completely flexed. Palmar pinch (Figure 1C): thumb pad to index pad and long finger pad. Key pinch (Figure 1D): thumb pad to index finger at the lateral middle phalanx (Mathiowetz et al., 1984; Visser et al., 2003; Goetz et al., 2012). Figure 1E shows the size of the electronic digital force dynamometer. Subjects were instructed to maintain identical arm postures throughout the entire test process. They could observe the output of pinch force from the computer monitor. Data were then acquired and processed with a computer that was installed with customized maximal voluntary isometric contraction and force reproduction testing programs (Kjyl Technologies, China).

### Maximal voluntary isometric contraction test

The subjects performed warm-up exercises before testing and were instructed to use all 3 pinch types with the maximal pinch force being applied on the dynamometer. Pinch types were displayed at random. Subjects were then instructed to exert and sustain a maximal grip force for 5 s, with the goal of reaching this maximum within 1 s, maintaining it for 3 s, and relaxing within 1 s. All pinch types were repeated twice for each, with the maximum over each types trial selected as this type pinch strength (Bao and Silverstein, 2005). A 3-min rest was allowed between the two tests to minimize fatigue’s impact.

### Force reproduction task

**FIGURE 2:**
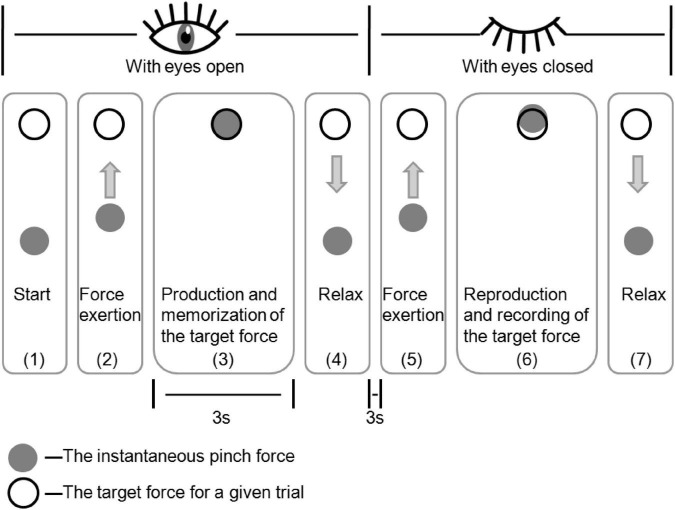
Sketch map showing output on the computer observed by monitor display, guiding subjects to the target force.

The subject’s accuracy in force reproduction tasks was measured to determine force sense. Each subject was informed of the test when watching the description presented on the computer monitor. Proprietary C++ software was utilized to display a black circle (which marked the target force in the specific task) on the screen to each subject. Afterward, the screen showed a gray dot, which indicated momentary pinch force (Figure 2). Each subject was asked to apply a pinch grip with the target force T for a 3-s period, and it was also requested that the applied force be memorized. Each subject was later allowed to relax and close both eyes by a verbal cue. At 3 s later, each subject was asked to repeat the prior force with the identical fingers in the absence of visual feedback. He or she hit the trigger with the contralateral hand when he or she suggested that the force applied was the same as the prior force; at this time, the force exerted (R) was recorded on the computer. Then, the subject could relax. This force reproduction test was conducted repeatedly until all subjects understood this process and were comfortable with the estimations. All subjects were asked to replicate 3 force levels [10, 30, 50% maximal voluntary isometric contraction (MVIC)] using all 3 pinch types, with 3 reproduction contractions being conducted at the respective force levels. Target forces were displayed at random. To prevent fatigue, subjects were allowed to rest for 30 s after every trial and for 2 min following 5 trials to enhance their attention during the testing process (Marini et al., 2017).

### Data analysis

There were four dependent variables for force sense errors: absolute error (AE), constant error (CE), variable error (VE) and the normalized absolute error (NAE). AE assesses overall error, whereas CE suggests error direction (over- or undershoot). VE indicates error variability after multiple trials, whereas NAE stands for performance accuracy level. For considering error as the MVIC percentage despite target force level, AE was calculated with the error normalized to the target force to obtain the normalized absolute error (NAE). These parameters were calculated using the following equation:


(1) $$AE=\frac{\sum_{i=1}^{3}\left|R_{i}-T\right|}{3},\left(i=1,2,3\right),$$

(2) $$CE=\frac{\sum_{i=1}^{3}\left(R_{i}-T\right)}{3},\left(i=1,2,3\right),$$

(3) $$VE=\sqrt{\frac{\sum_{i=1}^{3}{\left(R_{i}-\bar{R}\right)}^{2}}{3}},\left(i=1,2,3\right)$$

(4) $$\text{NAE}=\frac{\sum_{\text{i}=1}^{3}\left|{\text{R}}_{\text{i}}-\text{T}\right|}{3\cdot T}\times 100\%,\left(\text{i}=1,2,3\right)$$

where *R*_i_ is the reproduction force for the i-trial and *T* is the target force, and is the mean of the force reproduced in three trials.

### Statistical analyses

Mixed-model ANOVAs (2-way ANOVA with repeated measures) were used to assess the impact of the pinch type (tip, palmar, and key pinch) and sex (men or women) on MVIC; in this model, sex was selected to be the between-subject factor, whereas pinch type to be the within-subject factor. Mixed-model ANOVAs were also used to assess the impact of the pinch type (tip, palmar, and key pinch), force levels (10, 30, and 50% MVIC) and sex (men or women) on AE, VE and NAE; in this model, sex was selected to be the between-subject factor, whereas force level and pinch type to be the within-subject factor. Simple-effects analyses were conducted when interaction is observed, and main effects without interaction were compared *via* a *post hoc* least significant difference (LSD) test. Additionally, CE values were compared by one-sample *t*-test at every force level and pinch types to 0, for the sake of identifying trials when subjects produced excess or insufficient force. SPSS22.0 (IBM, Armonk, NY, USA) was adopted for statistical analysis. Results were displayed in a form of mean ± SD, with *P* < 0.05 indicating statistical significance.

## Results

### Maximal voluntary isometric contraction

**FIGURE 3:**
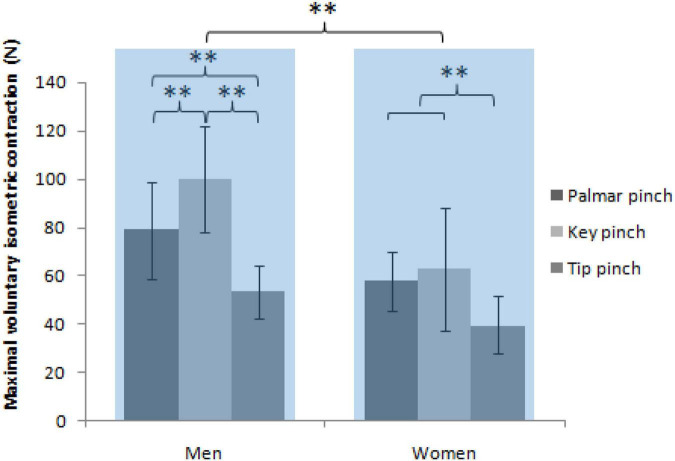
MVIC (average from groups) as a function of the sex and pinch types (^**^ = *P* < 0.01).

Mixed-model ANOVA was used to compare MVIC, revealing a significant interaction was observed between the sex and pinch types, *F*(2, 76) = 11.88, *P* < 0.001. Follow-up simple effects tests for the sex based on the pinch type revealed significantly higher MVIC of the men compared with women of palmar [men: 78.9 ± 4.5 N, women: 57.8 ± 2.8 N, *t* (114) = 4.35, *P* < 0.001], key [men: 100.1 ± 4.9 N, women: 62.6 ± 2.5 N, *t* (114) = 7.74, *P* < 0.001], and tip pinch [men: 53.1 ± 2.4 N, women: 38.7 ± 2.4 N, *t* (114) = 2.96, *P* = 0.004]. Follow-up simple effects tests for the pinch type by sex revealed a significant lower MVIC of the tip pinch compared with the palmar pinch [men: *t* (114) = 5.33, *P* < 0.001, women: *t* (114) = 3.95, *P* < 0.001] and key pinch [men: *t* (114) = 9.72, *P* < 0.001, women: *t* (114) = 4.94, *P* < 0.001) in both sex. Palmar pinch showed markedly decreased MVIC compared with the key pinch in men [*t* (114) = 4.39, *P* < 0.001] but not in women [*t* (114) = 1.00, *P* = 0.965; Figure 3].

### Absolute error

**FIGURE 4:**
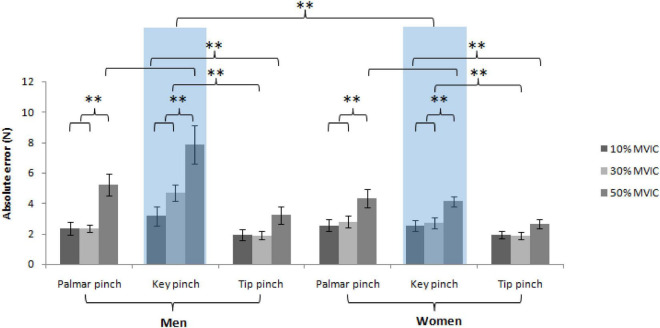
Absolute error assessing overall error in force reproduction against the sex, pinch types and force levels (^**^ = *P* < 0.01).

This work utilized mixed-model ANOVA for computing absolute error, revealing no significant interaction was observed between the sex, force levels and pinch types, *F*(4, 156) = 1.69, *P* = 0.155. But a significant interaction was observed between the sex and pinch types, *F*(2, 76) = 14.82, *P* < 0.001, and pinch types and force levels, *F*(4, 156) = 4.49, *P* = 0.002, but not between the sex and force levels, *F*(2, 76) = 2.26, *P* = 0.111. Subsequently, follow-up simple effects tests for the pinch types based on the sex revealed significantly higher absolute error values in men (5.2 ± 0.6 N) than in women (3.1 ± 0.2 N) of the key pinch [*t* (114) = 4.57, *P* < 0.001], but not of the tip [men: 2.3 ± 0.2 N, women: 2.2 ± 0.2 N, *t* (114) = 0.41, *P* = 0.681] and palmar pinch [men: 3.3 ± 0.3 N, women: 3.2 ± 0.3 N, *t* (114) = 0.18, *P* = 0.861]. Follow-up simple effects tests for the force level based on the pinch types revealed significantly lower absolute error of the tip pinch (men: 1.9 ± 0.3 N, women: 1.9 ± 0.2 N) compared with the key pinch (men: 4.7 ± 0.5 N, women: 2.7 ± 0.4 N) at 30% MVIC [*t* (114) = 3.41, *P* = 0.002]. There was a significant lower absolute error of the tip pinch (men: 3.2 ± 0.6 N, women: 2.6 ± 0.3 N) compared with the key pinch [men: 7.9 ± 1.3 N, women: 4.1 ± 0.3 N, *t* (114) = 5.66, P < 0.001] and palmar pinch [men: 5.2 ± 0.7 N, women: 4.3 ± 0.6 N, *t* (114) = 3.42, P = 0.002] at 50% MVIC. Follow-up simple effects tests for the pinch types based on the force level revealed significantly lower absolute error at 10% [palmar pinch: 2.5 ± 0.3 N, *t* (114) = 4.33, *P* < 0.001, key pinch: 2.8 ± 0.4 N, *t* (114) = 5.84, *P* < 0.001] and 30% [palmar pinch: 2.6 ± 0.2 N, *t* (114) = 4.12, *P* < 0.001, key pinch: 3.7 ± 0.4 N, *t* (114) = 4.24, *P* < 0.001] compared with 50% MVIC (palmar pinch: 4.8 ± 0.5 N, key pinch: 6.0 ± 0.7 N) of palmar and key pinch in both sex (Figure 4).

### Variable error

**FIGURE 5:**
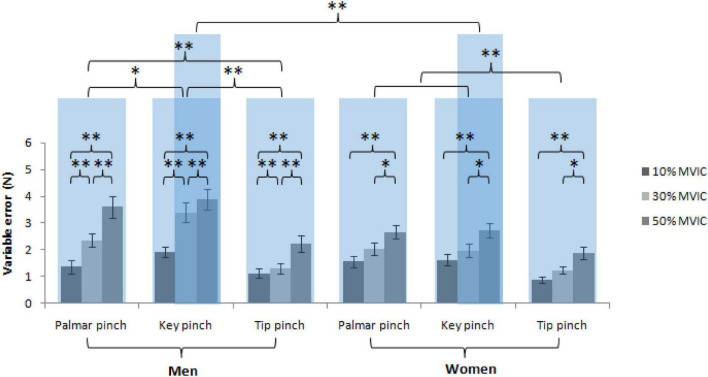
VE indicates the error variability after multiple trials and performance accuracy against sex, force levels and pinch types (^*^ = *P* < 0.05; ^**^ = *P* < 0.01).

Mixed-model ANOVA was used to compute variable error, revealing no significant interaction was observed between the sex, force levels and pinch types, *F*(4, 156) = 1.34, *P* = 0.260. Nonetheless, there was a significant interaction between sex and pinch types, *F*(2, 76) = 4.28, *P* = 0.017, sex and force levels, *F*(4, 156) = 3.95, *P* = 0.023, but there was no significant interaction between pinch types and force level, *F*(2, 76) = 1.47, *P* = 0.215. Subsequently, follow-up simple effects tests for the pinch types based on the sex revealed significantly higher variable error values in men than in women of the key pinch at 30% [men: 3.4 ± 0.4 N, women: 2.0 ± 0.2 N, *t* (114) = 2.64, *P* = 0.009] and 50% MVIC [men: 3.9 ± 0.4 N, women: 2.7 ± 0.3 N, *t* (114) = 3.58, *P* < 0.001], but not at 10% MVIC [men: 1.9 ± 0.2 N, women: 1.6 ± 0.2 N, *t* (114) = 0.48, *P* = 0.630]. Additionally, there was no significant different variable error of the tip and palmar pinch between men and women (all *P* > 0.05). Follow-up simple effects tests for the sex based on the pinch types revealed significantly lower variable error of the tip pinch [1.6 ± 0.1 N, *t* (114) = 3.86, *P* < 0.001] and palmar pinch [2.4 ± 0.2 N, *t* (114) = 3.87, *P* < 0.001] compared with the key pinch (3.1 ± 0.2 N) in men. There was a significant lower variable error of the tip pinch (1.3 ± 0.1 N) compared with the key pinch [2.1 ± 0.1 N, *t* (114) = 2.92, *P* = 0.011] and palmar pinch [2.1 ± 0.1 N, *t* (114) = 4.63, *P* < 0.001] in women. Follow-up simple effects tests for the sex based on the force level revealed significantly lower variable error at 10% [1.5 ± 0.1 N, *t* (114) = 3.75, *P* = 0.001] and 30% [2.4 ± 0.2 N, *t* (114) = 6.41, *P* < 0.001] compared with 50% MVIC (3.2 ± 0.2 N) in men. There was a significant lower variable error at 10% compared with 30% MVIC [*t* (114) = 2.65, *P* = 0.025] in men. There was a significant lower variable error at 10% [1.4 ± 0.1 N, *t* (114) = 3.18, P = 0.005] and 30% [1.8 ± 0.1 N, *t* (114) = 3.25, *P* = 0.004] compared with 50% MVIC (2.4 ± 0.2 N), but no significant different variable error between 10 and 30% MVIC in women [*t* (114) = 0.07, *P* = 1.000; Figure 5].

### Normalized absolute error

**FIGURE 6:**
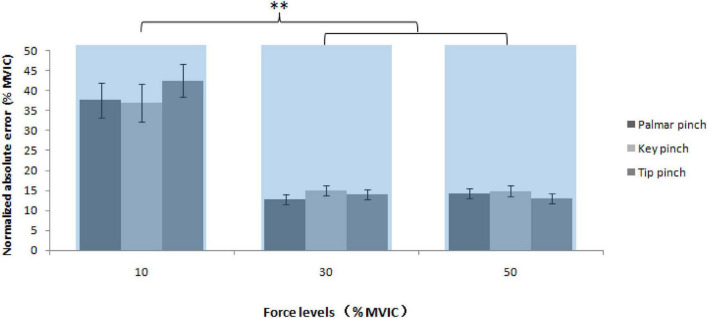
NAE to the maximal voluntary isometric contraction, as a function of pinch types and force levels (^**^ = *P* < 0.01).

Mixed-model ANOVA was used to compute the normalized absolute error, revealing no significant interaction was observed between the sex, force levels and pinch types, *F*(4, 156) = 0.15, *P* = 0.962, the sex and pinch types, *F*(2, 76) = 2.61, *P* = 0.080, the sex and force levels, *F*(4, 156) = 2.48, *P* = 0.091, and pinch types and force levels, *F*(2, 76) = 1.27, *P* = 0.284. Normalized absolute error between force levels was significantly different, *F*(1, 39) = 46.49, *P* < 0.001, but not the sex [men:19.6 ± 1.9% MVIC and women: 25.0 ± 1.9% MVIC, *F*(1, 39) = 4.02, *P* = 0.052] and pinch types [the palmar pinch: 21.6 ± 1.6% MVIC, key pinch: 22.3 ± 1.7% MVIC and tip pinch: 23.2 ± 1.6% MVIC, *F*(1, 39) = 0.55, *P* = 0.581]. There was a significant lower normalized absolute error values of the 30% MVIC [13.9 ± 0.9% MVIC, *t* (114) = 7.31, *P* < 0.001] and 50% MVIC [14.1 ± 1.2% MVIC, *t* (114) = 6.76, *P* < 0.001] compared with 10% MVIC (39.0 ± 3.5% MVIC; Figure 6).

### Constant error

**FIGURE 7:**
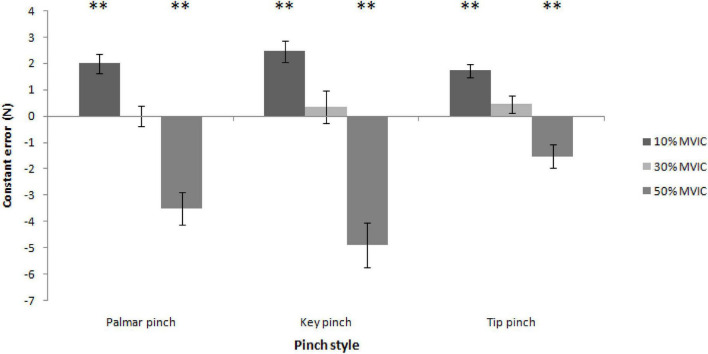
Constant error, the force of reproduction directionality of error, as a function of the pinch types and force levels (^**^ = *P* < 0.01).

Significantly higher constant error values were detected at force levels at 10%MVIC in men and women, respectively [all *t* (39) > 5.70, *P* < 0.01], while markedly decreased CE could be obtained at the force levels of 50% MVIC for men and women, respectively [all *t* (39) < -3.36, *P* < 0.01]. Moreover, direction estimations for men and women were most accurate for force levels at 30% MVIC [*t* (39) = 0.02–1.44, all *P* > 0.05; Figure 7].

## Discussion

### Maximal voluntary isometric contraction

Our present finding is concordant with previous studies (Mathiowetz et al., 1985; Mohammadian et al., 2015) that revealed that pinch force values significantly increased among male adults compared with female adults, along with decreased tip pinch compared with the palmar and key pinch in both sexes. Additionally, there was a significantly lower pinch force of the palmar pinch than the key pinch in men but not in women. This relationship of sex with pinch type suggested increased differences between strength capacity and key/palmar pinch among male adults compared with female adults. It is possibly associated with the increased utilization of thenar muscles in sports and different work types in male adults (Dempsey and Ayoub, 1996).

### Pinch-type effects on pinch force sense

Our results revealed no significantly different absolute error values between different pinch types at 10% MVIC. However, there was a significantly lower absolute error of the tip pinch compared to the key pinch at 30% MVIC and a the significantly lower absolute error of the tip pinch compared to the key and palmar pinch at 50% MVIC. Our results also revealed significantly lower variable error of the tip pinch than the key and palmar pinch in both men and women and a significantly lower variable error of the palmar pinch than the key pinch in men but not in women.

The difference in tip pinch (the thumb and index finger) and palmar pinch (the thumb, index, and long finger) is the number of fingers. The force vectors of each finger weakened when the digit numbers increased in the pinch grip. For example, when three fingers pinch, due to the different lengths of the middle finger and index finger, the latter should have increased joint angle, whereas that of the former should be reduced so that the two fingers are aligned to hold the object relative to the thumb. A change in the position of the fingers during the three-finger pinch, leading to a deviation from the optimal position of power generation, may affect the perception of power (Shih et al., 2003; Puh, 2010; Jahn et al., 2013; Kim et al., 2020). In conclusion, achieving an appropriate pinch grip necessitates the ability to accurately coordinate the fingers through a combination of sensation and force control, and as the number of fingers increases (palmar pinch), so does the difficulty associated with such pinch grip coordination (Kong et al., 2011; Kim et al., 2020).

The difference between the key pinch (index finger at lateral middle phalanx) and tip pinch (index pads) lies in the thumb pad to the other two pads. Hairless skin on the finger pad, a highly innervated region in the body, contains numerous cutaneous receptors that can sense skin stretching orientation and oriented pressure (Blanchard et al., 2011). It is well established that the tactile acuity of the finger pad is greater than that of the lateral skin of the finger (Gutierrez and Santos, 2020). Additionally, the corticospinal projection is especially dense for the finger pad (Gandevia and Kilbreath, 1990; Häger-Ross and Johansson, 1996; Macefield and Johansson, 1996; Macefield et al., 1996; Gutierrez and Santos, 2020). In summary, the finger pad clearly shows greater tactile acuity than the lateral skin of the finger, resulting in the tip pinch being more sensitive than the key pinch.

Considering ergonomics, approaches have been prepared to suit the best pinch types, thus maximizing the precise pinch grip strength generation and estimation. Choosing the tip pinch grip to accurately apply pinch grip force when a specific object can be held with a low force is necessary. Meanwhile, in the design of hand-held tools, or substances utilized in the assembly line (Byström et al., 1995), in sports (Chang et al., 2010), or to perform dental work (Dong et al., 2006), it is necessary to consider the relation of pinch type with pinch grip force sense.

### Sex effects on pinch force sense

Our results are inconsistent with the hypothesis that women reproduce key pinch force more accurately (absolute error) and consistently (variable error, at 30% and 50% MVIC) than men. However, there was no significant difference in the normalized absolute error between men and women.

Women reproduce key pinch force more accurately (absolute error) than men. However, there was no significant difference in the normalized absolute error between men and women. Previous studies have shown that proprioceptive sensors, such as Golgi tendon organs (GTOs), have high sensitivity to higher forces, whereas tactile sensors show higher sensitivity to low forces (Onneweer et al., 2013). GTOs within muscles can detect information related to interaction forces and muscle forces (McCloskey, 1978; Crago et al., 1982; Jami, 1992; Proske and Gandevia, 2012; Onneweer et al., 2013). GTOs facilitate the direct measurement of tendon strain, which can appropriately explain alterations of tendon forces at diverse loading levels and predict muscle-tendon complex length (Herter et al., 2021). The relationship between the force generated by a muscle and the discharge rate of the nearby GTOs has been reported to be linear (Crago et al., 1982). This is similar to a strain gauge (Golgi interorgan) attached to a deformable piece (muscle). The strain gauge will create a microstrain, directly proportional to the force applied. The overall resolution (NAE) maybe the same between men and women; the larger the measurement range (MVIC), the smaller the accuracy (more AE in men). Additionally, our results also show that subjects are not significantly different in sensitivity to detecting changes in their pinching force using different pinch types when the error is normalized to the maximal voluntary isometric contraction. These results are consistent with a prior study (Gandevia and Kilbreath, 1990; Kilbreath and Gandevia, 1993; Jones, 2003) regarding the relative perception of force, which can be scaled in line with muscle operation range.

Women reproduce key pinch force more consistently (variable error) at 30% and 50% MVIC than men, but there is no significantly different variable error at 10% MVIC between men and women. Variable error accounts for deviation near the average reproduction force, resulting from sensor uncertainty, such as sensor noise (Arnold and Docherty, 2006). Based on the previously mentioned research by Brothers, the finger pad reacts to low force levels, which occurs because of the low threshold of the SA-I mechanoreceptors (Brothers and Hollins, 2014). Testing of tactile sensitivity revealed no significantly different skin sensitivity in men compared with women (Weinstein, 1968). Thus, there was no significant difference in the variable error at low force levels (10% MVIC) between men and women.

This study showed that the difference between men’s and women’s force perception was interactively influenced by the pinch type (only the key pinch showed gender differences, but not the other pinch types) and force levels and was related to the selected dependent variables (absolute error, variable error or the normalized absolute error). This study, along with previous studies, shows that force performance in accuracy differences between sexes depends on numerous aspects, such as the design of experiments [force reproduction task (Bao and Silverstein, 2005) or force matching task (Song et al., 2006; Herring-Marler et al., 2014), target force [9.8 N (Bao and Silverstein, 2005), 5% MVIC (Herring-Marler et al., 2014) or 50% MVIC (Song et al., 2006)], dependent variables [absolute error, constant error, variable error, normalized absolute error, coefficients of variation (Bao and Silverstein, 2005) or root mean square error (Herring-Marler et al., 2014)], aging [19–63 (Bao and Silverstein, 2005), 30–79 (Herring-Marler et al., 2014) or 23.4 ± 3 (Song et al., 2006) years old], and joints [key pinch, palmer pinch (Bao and Silverstein, 2005), tip pinch (Herring-Marler et al., 2014) or knee (Song et al., 2006)]. When comparing the force sense of men and women, discussions should be made according to different conditions. Therefore, further research is required to understand the relationship between sex and force sense more clearly.

### Limitations

Our study has some limitations. For example, only healthy adult subjects with a mean age of 22.3 years were enrolled in this study. Therefore, the conclusions in the study may only be valid for the assessment of the pinch force sense in similarly aged and healthy adult. Thus, additional studies are needed to examine these relationships among other age groups.

## Conclusion

The results of our study revealed that women were more accurate (absolute error) or consistent (variable error) at detecting changes in their pinching force than men. However, no significant difference was found when the error normalized to the maximal voluntary isometric contraction (normalized absolute error). The difference in force sense between male and female adults was affected by the pinch type and force levels and was related to the selected dependent variables. Subjects are significantly more sensitive to the detection of alterations of pinch force by adopting tip pinch compared with key and palmar pinch with high forces (30 and 50% MVIC), but not with low forces (10% MVIC). Our observations and prior studies help understand the increased MSD rate among female adults used to guide the design of tools and interventional approaches for improving pinch grip force perception and reducing hand injury rates in both men and women.

## Data availability statement

The datasets presented in this study can be found in online repositories. The names of the repository/repositories and accession number(s) can be found below: osf.io/3rtvx/.

## Ethics statement

This work gained approval by the Ethics Review Board of Renmin University of China (reference number: 2021057). The patients/participants provided their written informed consent to participate in this study. Written informed consent was obtained from the individual(s) for the publication of any potentially identifiable images or data included in this article.

## Author contributions

LL: conceptualization, data curation, software, writing – original draft and review, and editing. YXL: conceptualization, methodology, funding acquisition, supervision, and writing – review and editing. PJ: data curation and writing – original draft. SW, WW, and YL: data curation and software. All authors contributed to the article and approved the submitted version.
